# Eph/ephrin-mediated immune modulation: a potential therapeutic target

**DOI:** 10.3389/fimmu.2025.1539567

**Published:** 2025-04-22

**Authors:** Konstantinos Giannopoulos, Ioannis Karikis, Chad Byrd, Georgios Sanidas, Nora Wolff, Maria Triantafyllou, Gabriele Simonti, Robinson Vidva, Ioannis Koutroulis, Stamatios Theocharis, Panagiotis Kratimenos

**Affiliations:** ^1^ National and Kapodistrian University of Athens School of Medicine, Athens, Greece; ^2^ Sheikh Zayed Institute for Pediatric Surgical Innovation, Children's National Hospital, Washington, DC, United States; ^3^ Department of Pediatrics, George Washington University, School of Medicine and Health Sciences, Washington, DC, United States

**Keywords:** skin cancer, skin cancer therapy, Eph/ephrin receptor system, melanoma, basal cell carcinoma, squamous cell carcinoma

## Abstract

Eph/ephrin signaling, a complex network of cell-cell interactions, plays a pivotal role in regulating various biological processes, including cell migration, proliferation, and adhesion. Dysregulation of this signaling pathway has been implicated in various types of cancer. In skin cancers such as squamous cell carcinoma, basal cell carcinoma, and malignant melanoma, Eph/ephrin signaling promotes tumor invasion and metastasis. Aberrant expression of Eph receptors and ephrin ligands can lead to increased cell motility, reduced cell adhesion, and enhanced angiogenesis. Furthermore, Eph/ephrin signaling can significantly impact the tumor microenvironment by modulating the infiltration and activation of immune cells, particularly T cells. Dysregulated Eph/ephrin expression can impair immune surveillance mechanisms, leading to immune evasion and tumor progression. For instance, certain ephrin ligands can inhibit T-cell activation and promote immunosuppressive conditions within the tumor microenvironment. Targeting Eph/ephrin signaling offers a promising therapeutic approach to combating skin cancer metastasis. By disrupting these signaling pathways, tumor cell invasion, angiogenesis, and immune evasion can be inhibited. This could lead to improved therapeutic outcomes for patients with skin cancer.

## Introduction

Skin cancer is among the most prevalent malignancies worldwide, with squamous cell carcinoma (SCC), basal cell carcinoma (BCC), and malignant melanoma being the most common forms (1). Melanoma is the most aggressive and deadly form of skin cancer. The mainstay of treatment for skin cancer is surgical excision (2). For non-melanoma skin cancer, such as BCC and SCC, excision of the tumor with a margin of healthy tissue is usually sufficient. For melanoma, treatment involves wide local excision, sometimes with lymph node dissection. In cases of unresectable tumors and metastasis, adjuvant therapies such as radiation, chemotherapy, immunotherapy, or targeted therapy may be implemented (3).

Like most cancer types, the pathogenesis and progression of skin cancer involves complex interactions between tumor cells and their microenvironment, wherein signaling pathways are dysregulated to promote expansion and spread of the tumor. One such signaling pathway is the Eph receptor and ephrin ligand family, vital for many cell functions such as cellular adhesion, migration, proliferation, and differentiation (4).

Eph receptors represent the largest family of receptor tyrosine kinases (RTKs), consisting of fourteen receptors that are classified into two subgroups: EphA and EphB, which interact with ephrin-A and ephrin-B ligands, respectively (4–6).

The Eph receptor family was first identified in 1987 during a screen for new oncogenic tyrosine kinases. The term “Eph receptor” originates from the ‘erythropoietin-producing hepatocellular carcinoma cell line,’ the cell line from which their cDNA was initially isolated. Since then, an entire family of receptors and ligands has been identified and categorized based on sequence homology. Despite their numerous functions across almost all tissues, these receptors remain among the least researched RTK families (4–6).

Ephrins and Eph receptors are crucial in cancer development, influencing cell proliferation, adhesion, migration, and angiogenesis (7). Their signaling is bidirectional—both forward and reverse signaling can either suppress or promote tumor growth, depending on the cancer type. This variability is due to the unique roles Eph/ephrin signaling plays in different tissues and contexts. For instance, the same pathway might inhibit tumor progression in one neoplasm but facilitate it in another, demonstrating the complexity of their role in oncogenesis (4, 7).

Eph/ephrin signaling has been extensively studied in neuronal development, but emerging evidence highlights its significant role in various cancers, including skin cancer. This review aims to provide an overview of Eph/ephrin signaling in skin cancer, focusing on SCC, BCC, and malignant melanoma and its relevance as a therapeutic target (**Table 1**).

**Table 1:** Summary of the key distinctions between squamous cell carcinoma, basal cell carcinoma, and malignant melanoma.

Feature	Squamous Cell Carcinoma (SCC)	Basal Cell Carcinoma (BCC)	Malignant Melanoma
Clinical Features	- Scaly, erythematous plaques or nodules - Can ulcerate - Common on sun-exposed areas (e.g., face, hands) - May metastasize (less common)	- Pearly, flesh-colored papules with telangiectasia - May ulcerate (“rodent ulcer”) - Rarely metastasizes but locally invasive	- Asymmetric, irregular borders, color variegation - Rapidly growing pigmented lesion - Can arise in sun-exposed or non-exposed areas - High potential for metastasis
Diagnosis	- Biopsy: Atypical keratinocytes invading dermis - Immunohistochemistry (IHC): p53, CK5/6, or SCC markers	- Biopsy: Palisading nuclei and basaloid cells - IHC: Ber-EP4, CK19	- Biopsy: Atypical melanocytes in epidermis and dermis- IHC: S100, HMB-45, Melan-A - Molecular studies (e.g., BRAF mutations)
Treatment	- Excision or Mohs surgery - Radiation for advanced cases - Systemic therapy for metastatic disease (e.g., immune checkpoint inhibitors)	- Excision or Mohs surgery - Rarely requires systemic treatment - Topical therapies for superficial lesions (e.g., imiquimod)	- Wide excision with sentinel lymph node biopsy - Targeted therapy (e.g., BRAF/MEK inhibitors) - Immunotherapy (e.g., anti-PD1, CTLA-4 inhibitors)
Prognosis	- Good if caught early - Worse with invasion or metastasis	- Excellent, with minimal risk of metastasis - Recurrence is common without complete excision	- Variable: poor with metastasis - 5-year survival depends on stage: Early stage: ~90%, Advanced stage: ~20-30%

## Structure of Eph receptors

**Figure 1:**
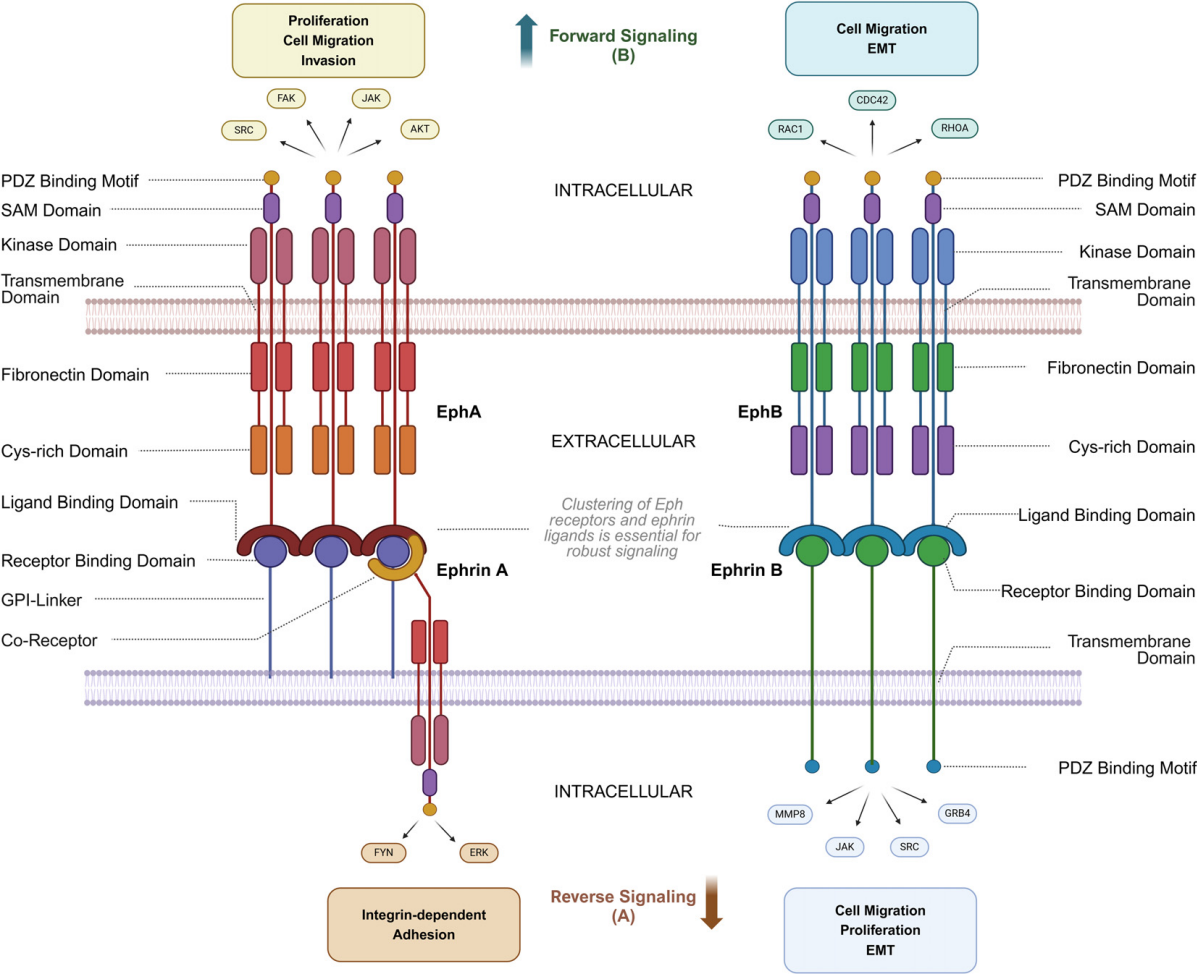
Structure and Interactions of Eph Receptors and Ephrin ligands. This diagram illustrates the bidirectional signaling pathways of Eph receptors and ephrin ligands, focusing on structural domains and associated signaling molecules. **(A)** Reverse signaling through ephrin ligands (ephrin-A and ephrin-B) is mediated by molecules such as FYN, ERK, JAK, SRC, and GRB4, regulating processes like integrin-dependent adhesion, cell migration, proliferation, and epithelial-mesenchymal transition (EMT). **(B)** Forward signaling through Eph receptors (EphA and EphB) involves AKT, SRC, FAK, and JAK, facilitating proliferation, cell migration, and invasion. Key structural domains, including the receptor-binding domain, ligand-binding domain, kinase domain, and PDZ binding motif, are shown, along with intracellular and extracellular regions critical for signaling. Cytoskeletal regulators such as CDC42, RAC1, and RHOA further modulate cell migration and EMT. Notably, robust Eph-ephrin signaling requires higher-order clustering of Eph receptors and ephrin ligands on opposing cell membranes, and because ephrin-A ligands are GPI-anchored, co-receptors are often necessary for strong reverse signaling into the ephrin-expressing cell. Parts of this figure were generated using BioRender.

Eph receptors share a conserved structure, including an extracellular ligand-binding domain, a cysteine-rich region, fibronectin type III domains, a transmembrane region, and an intracellular juxtamembrane domain, followed by a kinase domain, SH2 domain, SAM domain, and a PDZ-binding site (8) (**Figure 1**). Upon ligand binding to the corresponding Eph receptor and clustering of Eph/ephrin complexes, phosphorylation of key tyrosine residues leads to further activation of signaling pathways (9). Unlike other RTKs, Eph receptor signaling is primarily mediated through pathways involving Rho and Ras GTPases, FAK, JAK-STAT, and PI3K (10). Ephrin ligands trigger forward signaling, while reverse signaling, although less understood, also plays a role. Eph receptor signaling is complex, often requiring clustering of receptors for efficient activation, and can occur through trans or cis interactions. Eph receptors can influence oncogenic pathways, such as EphA2 interacting with ErbB2 to enhance cell motility and proliferation. Recent studies highlight their roles in exosome-mediated communication and ligand-independent signaling, particularly in the context of cancer (11, 12).

Reverse signaling has been demonstrated for both ephrin-A and ephrin-B ligands. Ephrin-A reverse signaling is mediated by transmembrane co-receptors—since ephrin-A lacks an intrinsic intracellular domain—with suggested involvement of neurotrophin receptors such as tropomyosin receptor kinase B (TrkB) and p75 neurotrophin receptor (p75NTR), as well as the Ret receptor tyrosine kinase. Conversely, ephrin-B reverse signaling is achieved through the phosphorylation of tyrosine residues in the linker region connecting the transmembrane domain to the PDZ-binding domain (13).

Ephrin-A ligands rely on these transmembrane co-receptors to mediate reverse signaling due to their GPI-anchored structure. For instance, p75NTR acts as a critical co-receptor in neural systems by forming complexes within caveolae, which facilitate cytoskeletal remodeling during axon guidance. Lim et al. (2008) demonstrated that p75NTR colocalizes with ephrin-A in retinal axons and is essential for Fyn kinase activation upon EphA binding, driving axon repulsion in the superior colliculus (14). This interaction is spatially regulated by lipid raft microdomains, which promote the clustering of ephrin-A/p75NTR complexes and enhance downstream RhoA/ROCK signaling.

Eph receptors and ephrin ligands exhibit complex, overlapping binding patterns that contribute to diverse signaling outcomes in different cellular contexts. Unlike many receptor-ligand systems that follow strict specificity, Eph receptors can bind multiple ephrins with varying affinities, and vice versa. For example, EphA2 can interact with both ephrin-A1 and ephrin-A2, while EphB4 can engage ephrin-B2 and ephrin-B3, leading to context-dependent signaling cascades. This cross-family binding introduces significant challenges in designing targeted therapies, as inhibition of one Eph-ephrin interaction may inadvertently affect parallel signaling pathways critical for normal cellular function.

## Ephrins in keratinocyte biology and epidermal homeostasis

Keratinocytes are the predominant cell type in the epidermis and rely on tightly regulated signaling pathways to maintain epidermal homeostasis. Eph receptors and ephrins are expressed in keratinocytes and control their proliferation, differentiation, and cell-to-cell adhesion (15). Studies have demonstrated that Eph/ephrin signaling regulates fundamental biological processes, including cell migration, myofibroblast activation, and tissue remodeling (15–17).

EphA4, EphB4, ephrin-A3, and ephrin-B1 are detected in various structural components of the skin, including hair follicles, sebaceous glands, and sweat glands. EphA receptor-mediated activation, particularly EphA1-EphA2 interactions, enhances the expression of desmosomal cadherin desmoglein 1, a key player in reinforcing cell-cell adhesion and keratinocyte differentiation. Moreover, ephrin-B signaling can promote keratinocyte differentiation and inhibit integrins and cell cycle regulators. Given these roles, Eph/ephrin signaling is emerging as a critical regulator of keratinocyte biology, which is disrupted in skin malignancies (18–21).

## Eph/ephrin signaling in angiogenesis and oncogenesis of skin cancers

Eph/ephrin signaling plays a significant role in tumor angiogenesis, an essential process for cancer progression. Eph receptors such as EphA1/A2, through interactions with ephrin-A1, regulate endothelial cells and their supporting structures to promote vascular growth. EphB2 enhances angiogenic signaling by affecting VEGF receptor endocytosis and regulating vascular endothelial growth (22). In various cancers, including colorectal cancer, the overexpression of EphB4 in tumor cells has been linked to increased tumor vascularization (23). Moreover, EphB2 signaling is crucial in tumor growth by controlling endothelial adhesion and migration, which are vital components of angiogenesis. Although these mechanisms are well-studied in other cancers, their specific roles in the angiogenesis of skin carcinoma are still under investigation, with preliminary findings suggesting that targeting Eph receptors could potentially curb the invasive growth (24).

## Eph/ephrin signaling and immune modulation in skin cancer

**Figure 2:**
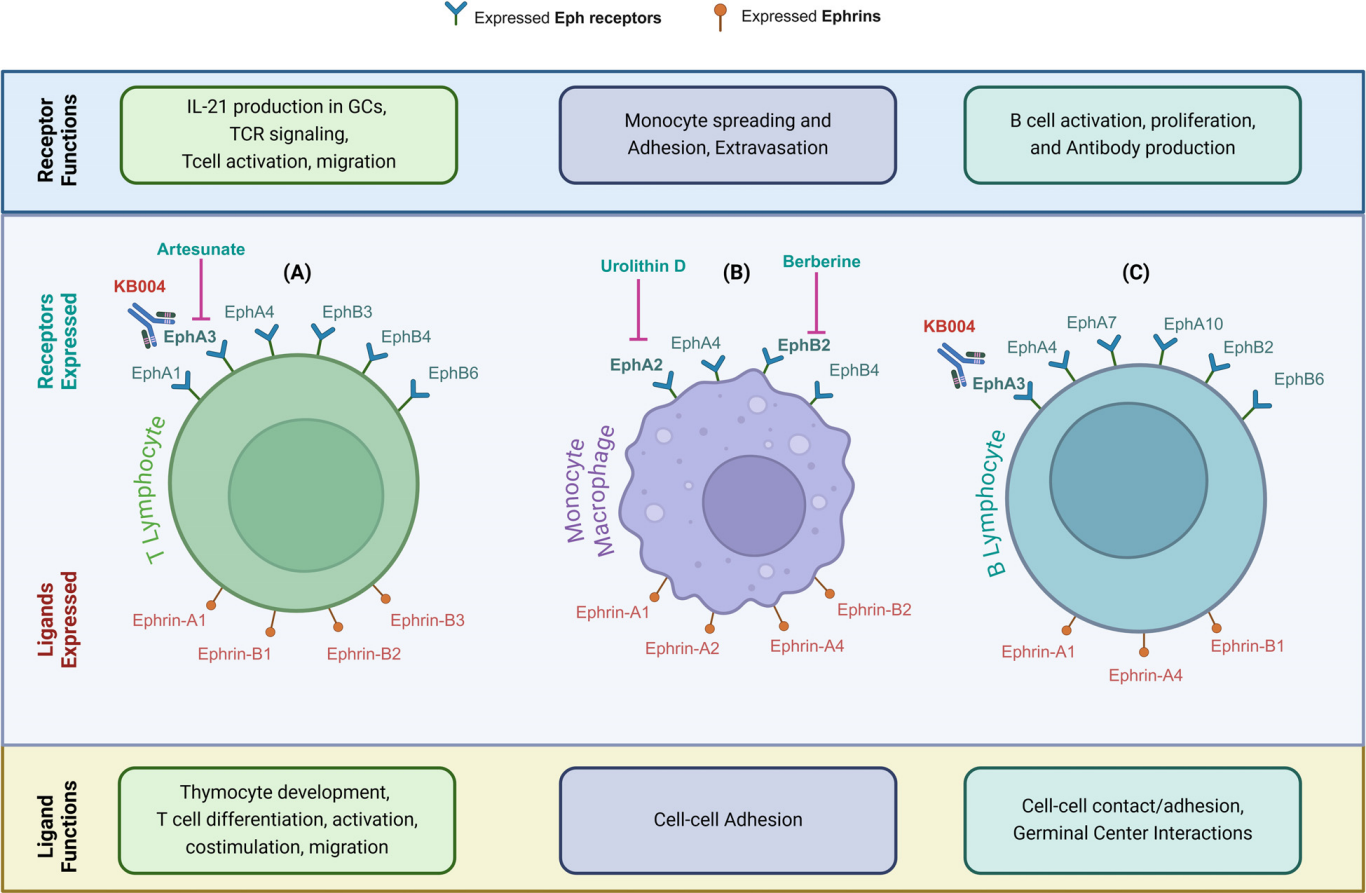
Eph Receptors and Ephrin Ligands Modulate Immune Cell Function. **(A–C)** Eph receptors and ephrin ligands expressed on immune cells—highlighting their distinct roles in immune regulation, including development, activation, adhesion, and functional interactions. **(A)** In T lymphocytes, Eph receptors mediate IL-21 production in germinal centers (GCs), T-cell receptor (TCR) signaling, T-cell activation, and migration. Ephrin ligands mediate thymocyte development, T-cell differentiation, activation, co-stimulation, and migration. **(B)** In monocytes/macrophages, Eph receptors mediate monocyte spreading, adhesion, and extravasation, while ephrin ligands mediate cell-cell adhesion. **(C)** In B lymphocytes, Eph receptors mediate B-cell activation, proliferation and antibody production, and ephrin ligands mediate cell-cell contact/adhesion and germinal center interactions. Therapeutic targeting of these pathways in malignant cells may also impact beneficial immune cell responsiveness, highlighting the need for strategic design to avoid antagonistic effects. Parts of this figure were generated using BioRender.

Ephrins and their receptors, particularly EphA and EphB receptors, play crucial roles in the development and activation of both the innate and adaptive immune systems (25, 26). These receptors are widely expressed across various immune cell types, including monocytes, macrophages, dendritic cells, platelets, and B and T lymphocytes, where they regulate essential immune functions (27, 28) (**Figure 2**). Ephrins are integral to both the physiological development of the immune system and pathological processes such as cancer and atherosclerosis. Due to their significant involvement in immune regulation and cancer progression, it is possible that ephrins also affect immune infiltration within tumors and, thus, the tumor microenvironment (TME) (29).

The immune system, particularly adaptive immune cells, is critical in recognizing and eliminating cancer cells. T cells are essential for effective anti-tumor immunity, recognizing tumor-specific antigens and initiating a cascade of immune responses, including recruitment of cytotoxic cells and cytokine production (30). However, tumors can evade immune surveillance by various mechanisms, such as downregulating antigen presentation, inducing immunosuppressive microenvironments, and directly targeting T cells. Eph/ephrin signaling contributes to the regulation of immune responses, including T-cell activation and migration (10). Ephrin-A1, for example, can inhibit T-cell activation and promote T-cell tolerance, while ephrin-B ligands can enhance T-cell responses and promote inflammation (27, 28). Eph/ephrin signaling plays a dual role in cancer progression, influencing both tumor cell behavior and the immune landscape within the tumor microenvironment. In malignant cells, EphA2 activation promotes invasion, migration, and metastasis via PI3K/Akt and MAPK signaling (31). Conversely, Eph receptors are also expressed on infiltrating immune cells, where they regulate T-cell activation, macrophage polarization, and dendritic cell function. For example, EphA2 on T cells has been implicated in modulating T-cell trafficking and immune synapse formation, which may have important implications for anti-tumor immunity (32). Additionally, EphB4-ephrin-B2 signaling has been shown to influence tumor-associated macrophage polarization, shifting the immune balance toward a more immunosuppressive phenotype (33). This functional divergence between tumor and immune cells highlights the challenge of developing Eph-targeted therapies that selectively inhibit oncogenic signaling without impairing anti-tumor immune responses.

A recombinant, de-fucosylated human immunoglobulin G1κ (IgG1κ) therapeutic monoclonal antibody, KB004, developed by KaloBios Pharmaceuticals (California, USA), demonstrated significant antitumor activity in solid tumor models by disrupting EPHA3-positive tumor stroma and vasculature (34). In addition, KB004 induced apoptosis and triggered antibody-dependent cellular cytotoxicity (ADCC) on leukemic blasts and tumor stem cells across a spectrum of leukemias, including acute myeloid leukemia, chronic myeloid leukemia, chronic lymphocytic leukemia, myelodysplastic syndromes, and myeloproliferative disorders (34).

## Eph receptors in thymocyte development and TCR signaling

Eph receptors play a critical role in regulating thymocyte development and T-cell modulation, with significant implications for T-cell receptor (TCR) signaling (35, 36). Within the thymus, EphA and EphB receptors contribute to the structural organization and function of the thymic cortex, essential for thymocyte maturation and survival (37). EphA4 and EphB6 are pivotal in these processes, as demonstrated in knockout studies where their absence led to reduced thymocyte counts and impaired peripheral T-cell populations. EphB receptors are particularly influential in TCR signaling, acting as co-stimulatory molecules that modulate T-cell activation (38). Activation of EphB receptors in T cells enhances responses to suboptimal TCR ligation, promoting proliferation, interferon-γ production, and cytotoxic T-cell activity, mediated through the upregulation of p38 and p42/44 MAP kinase pathways (39). However, EphB receptors exhibit a dual function; they can also blunt the effects of strong TCR signaling, curbing excessive interleukin-2 secretion and preventing apoptosis. This regulatory balance suggests that EphB receptors support the positive selection of thymocytes while protecting against overactive immune responses that could lead to autoimmunity.

EphA receptors, on the other hand, modulate thymocyte responses to antigens by inhibiting the negative selection of self-reactive thymocytes (35). Through interactions with ligands like ephrin-A1, EphA signaling can suppress interleukin-2 secretion and apoptosis in response to strong TCR activation, thereby influencing thymocyte maturation and T-cell development. These combined roles of Eph receptors underscore their complex involvement in guiding T-cell development and maintaining immune homeostasis (35, 40).

Dysregulation of Eph/ephrin signaling in skin cells can contribute to tumorigenesis and immune evasion. For instance, altered expression of Eph receptors and ephrin ligands in skin cancer cells may impact the infiltration and function of T cells within the TME (41). This could lead to impaired immune surveillance and increased tumor growth and metastasis.

## Ephrins and malignant melanoma

Malignant melanoma is one of the most aggressive forms of skin cancer, responsible for the majority of skin cancer-related deaths. Melanoma progression involves the transition from benign nevi to malignant lesions, characterized by radial and vertical growth phases, the latter being associated with increased metastatic potential (42). The development of malignant melanoma involves a complex interplay of environmental and genetic factors.

Recent insights have expanded our understanding of how Eph/ephrin signaling contributes to malignant cell proliferation, particularly in melanoma (43, 44). EphA3, for instance, is upregulated in melanoma and contributes to its malignancy by activating key pathways such as ERK1/2 and p38 MAPK. This signaling is negatively regulated by microRNA-3666, presenting an opportunity for targeted therapeutic interventions to curb melanoma progression (45).

Resistance to immunotherapy is a well-recognized challenge in the context of melanoma (46). Treatments with immune checkpoint inhibitors such as nivolumab and pembrolizumab, which target the PD-1/PD-L1 axis, are cornerstones of advanced melanoma therapy. However, not all tumors respond effectively to these therapies, and the presence of cytotoxic CD8+ T cells in the TME has been identified as a critical factor in determining therapy success (47).

A study by Markosyan et al. identified EphA2 as a candidate driver of immune suppression in pancreatic adenocarcinoma. Deletion of EphA2 in tumor cells sensitized these tumors to immune checkpoint inhibitors by increasing CD8+ T-cell infiltration, underscoring EphA2 blockade as a potential therapeutic target for enhancing anti-tumor immunity (48). Given the established role of EphA2 in melanoma progression, it is reasonable to hypothesize that similar mechanisms may be at play in melanoma, where EphA2 overexpression may drive immunosuppression and metastasis.

Given the central role of EphA2 in melanoma progression and its involvement in immune suppression, EphA2-targeted therapies hold promise not only for inhibiting metastatic spread but also for enhancing the efficacy of immune checkpoint inhibitors. Future studies should focus on elucidating the mechanisms by which EphA2 influences the immune landscape of the TME, as well as developing EphA2 inhibitors to overcome resistance to current therapies.

Beyond immune evasion, the role of Eph receptors, particularly EphA2, has gained considerable attention for contributing to other mechanisms of melanoma progression as well. EphA2 expression is higher in metastatic melanoma cells than in primary lesions (49). Silencing EphA2 in metastatic melanoma cells reduces their viability, migration, and invasiveness, indicating the critical role of EphA2 as a survival factor in melanoma. Furthermore, EphA2 has been implicated in resistance to targeted therapies for melanoma, such as vemurafenib, a BRAF inhibitor. Suppression of EphA2 can reduce the activation of downstream pathways like Akt and ERK, thus limiting melanoma progression and proving to be a potential therapeutic target (50).

Interestingly, ephrin-A1, a ligand for EphA2, was also upregulated in 67% of metastatic melanomas and 43% of progressed primary melanomas and has been shown to promote tumor angiogenesis through extensive interactions with endothelial cells. The EphA2/ephrin-A1 axis, influenced by inflammatory cytokines such as TNF-α and IL-1β, plays a crucial role in melanoma’s metastatic behavior. However, research suggests that ephrin-A1’s role in metastasis may differ depending on the cellular context, with some studies indicating that ephrin-A1 can inhibit metastatic behavior (51, 52).

Zhang et al. demonstrated that in cases of BRAF inhibitor-resistant melanoma, the metastatic potential of the tumor was significantly influenced by EphA2 signaling. They reported that non-canonical EphA2 signaling promoted melanoma cell invasion, survival under shear stress, adhesion to endothelial cells under continuous-flow conditions, increased permeability of endothelial cell monolayers, and stimulated melanoma transendothelial cell migration through complex mechanisms. Most importantly, they found that inhibiting EphA2 reduced invasion and blocked melanoma cell-endothelial cell interactions. These findings suggest that EphA2 inhibition could potentially improve prognosis in cases where EphA2 signaling drives melanoma metastasis (53).

Moreover, Eph receptors other than EphA2, such as EphB4 and its ligand ephrin-B2 and EphB6, have been shown to contribute to melanoma progression. EphB6 expression is reduced in metastatic melanoma, and its downregulation correlates with metastatic melanoma cell lines and, thus, poor prognosis. EphB4, on the other hand, promotes melanoma cell adhesion but appears to have a more complex role in migration, potentially influenced by interactions with macrophages (54).

Broggini et al. demonstrated that EphB4/ephrin-B2 communication could be a potential therapeutic target for melanoma. Their study found that this axis was key in promoting bone metastasis in an *in vivo* melanoma model (55).

The role of ephrin-B2 in melanoma goes beyond promoting EphB signaling; it also plays a role in tumor progression through its association with integrins. Specifically, endogenous ephrin-B2 expressed in melanoma cells has been found to interact with β1-integrins, which facilitates cell adhesion and migration. This interaction underscores the importance of reverse signaling and the crosstalk between the ephrin and integrin pathways in enhancing the invasive and migratory capabilities of melanoma cells (40, 56).

To summarize, these findings suggest that Eph/ephrin signaling plays a critical role in melanoma progression, metastasis, and resistance to immunosurveillance and therapy. EphA2 and EphB4 represent promising therapeutic targets, while the loss of EphB6 may serve as a prognostic marker for melanoma progression.

## Squamous cell carcinoma and Eph/ephrin signaling

Primary SCCs are highly likely to recur and metastasize (57). The molecular mechanisms driving SCC progression are only partially understood (58). Despite its high incidence, therapeutic management of metastatic SCC remains challenging due to the absence of effective targeted therapies (59–61). Given the involvement of the regulatory interplay between the Eph/ephrin signaling and the immune system and considering the Eph/ephrin involvement in the malignant transformation of SCC, we highlight this Eph/ephrin-immune regulatory interplay as a potential opportunity for therapeutic intervention for SCC.

Eph/ephrin signaling, mainly through EphB2, has been implicated in SCC progression (62). The expression of EphB2 in SCC has been associated with clinicopathological features like cancer staging and disease-free survival. Also, EphB2 overexpression in SCC is associated with poor prognostic factors, such as lymph node metastasis and high histological grade. Knockdown studies of EphB2 in SCC models show a significant reduction in tumor growth and invasion, suggesting a crucial role for EphB2 in promoting SCC tumorigenicity. In addition, Li et al. inhibited EphB2 *in vitro* and in xenograft models of SCC. Their results showed that EphB2 inhibition markedly reduced skin cancer cell proliferation, induced apoptosis, altered the cell cycle, and inhibited cell invasion and migration (63). EphB2 is both a biomarker for SCC and a driver of early tumor progression toward invasive disease.

Additionally, a recent study has highlighted EphB4 and its ligand ephrin-B2 as essential factors in SCC angiogenesis, with their inhibition leading to tumor regression. Specifically, Bhatia et al. demonstrated that EphB4 inhibition in combination with radiotherapy achieved reprogramming of the tumor immune microenvironment in patients with head and neck SCC (33).

Another Eph receptor implicated in head and neck SCC is EphA2. Downstream signaling of EphA2 includes the Akt-mTORC1, Raf-MEK-ERK, and Pyk2-Src-ERK pathways (64). Upregulation of EphA2 in head and neck SCC promotes lymph node metastases and has been associated with higher clinical stage of the tumor and reduced patient survival (65).

These findings suggest that targeting the Eph/ephrin pathway in SCC may provide a novel therapeutic avenue, particularly for patients with advanced or unresectable tumors.

## Ephrins and basal cell carcinoma

BCC is the most common type of skin cancer, characterized by slow growth and a low metastatic rate. Despite its generally indolent nature, BCC can exhibit local invasive behavior, posing therapeutic challenges in advanced or recurrent cases. Unraveling the molecular mechanisms underlying this invasive potential remains critical for improving treatment strategies (66, 67).

Recent studies have highlighted the downregulation of specific Eph receptors, particularly EphA7, in BCC, with hypermethylation of CpG islands in its promoter region being a key mechanism of this suppression (68). This downregulation was found in a significant portion of BCC samples (44.4%), with hypermethylation present in 90% of these cases, while EphA7 was positively expressed in normal basal cells and benign skin lesions. Although BCC is generally less prone to metastasis than other skin cancers, the reduced expression of EphA receptors like EphA7 might still contribute to local invasive behavior. Interestingly, other potential mechanisms, such as miRNA regulation, could also play a role in EphA7 loss in BCC. These findings suggest that targeting Eph receptor signaling, particularly in cases with downregulated expression, could offer new therapeutic avenues for limiting local invasiveness in BCC. However, its relevance in metastatic potential remains under investigation (68). Further research on Eph receptors as therapeutic targets could potentially shift the understanding of BCC progression.

## Natural compounds targeting Eph/ephrin signaling

The promising potential of Eph/ephrin-targeted treatments for skin cancer management is supported by the therapeutic properties of natural compounds that target Eph/ephrin signaling. Artesunate (ART), a derivative of artemisinin sourced from the sweet wormwood plant, has shown notable anti-cancer effects against choroidal melanoma (CM). Studies have demonstrated that ART can inhibit the proliferation and migration of CM cells by suppressing the expression of ephrin-A3 and downregulating key pathways such as Stat3/Akt. ART treatment significantly reduced tumor growth in xenograft tumor models, underscoring its potential as a therapeutic option for CM (69).

Berberine, an alkaloid in plants like *Coptis chinensis*, has exhibited anti-cancer properties across various tumor types, including breast cancer. While its specific effect on skin cancer has not been extensively studied, the mechanisms identified in other types of cancer are relevant. Berberine has been found to inhibit cell proliferation and migration, potentially through targeting proteins like ephrin-B2, and modulates critical cancer-associated pathways, including apoptosis and angiogenesis. These effects suggest that berberine could be an effective agent against skin cancers and merit further exploration (70).

Ellagitannins, polyphenolic compounds from fruits such as pomegranates and berries, also exhibit anti-cancer effects through their colonic metabolites, particularly urolithin D. Although primarily investigated in the context of prostate cancer, urolithin D has been shown to selectively inhibit phosphorylation of EphA2, a key protein in tumor progression. This selective action on the Eph/ephrin system indicates potential therapeutic applications that could extend to skin cancer treatment (71).

These examples of natural compounds illustrate the substantial potential of plant-derived substances to target various Eph/ephrin-related tumorigenesis pathways, including cell proliferation, migration, and critical signaling mechanisms. While compounds such as artesunate have been directly tested in melanoma models, others like berberine and urolithins have shown promising results in different cancers, suggesting further specific research on their effects on skin cancers. Continued studies, including clinical trials, are essential to fully understand their therapeutic efficacy, safety, and possible integration into skin cancer treatment regimens.

## Perioperative strategies for management of metastatic skin cancer

A combination therapy integrating Eph/ephrin inhibitors with immunotherapy could revolutionize perioperative management of skin cancer, potentially preventing metastasis. Although skin cancer surgery is highly effective, there is obvious value in exploring adjuvant therapies to address any microscopic residual disease that may contribute to recurrence or metastasis. By targeting Eph/ephrin signaling, we can disrupt tumor cell migration and invasion, limiting their ability to spread to distant sites. While immunotherapy can bolster the immune system’s capacity to recognize and eliminate any residual cancer cells, pairing it with Eph/ephrin-targeted treatments could help further reduce the risk of recurrence. This synergistic approach offers a promising strategy to improve patient outcomes and reduce the burden of skin cancer.

A similar synergistic effect could be achieved even through targeting different aspects of the Eph/ephrin system in and of itself. For instance, inhibiting ephrin-A1, which promotes tumor cell migration and invasion, could limit the spread of cancer cells. Conversely, targeting EphB receptors, which can regulate immune cell function, may enhance anti-tumor immunity. Taking advantage of the multifaceted roles of Eph/ephrin signaling by following a dual approach with different Eph/ephrin inhibitor combinations could synergistically suppress tumor growth and metastasis.

The complex interplay between Eph/ephrin signaling and the TME presents a promising therapeutic avenue for skin cancer. By targeting key Eph receptors and ephrin ligands, we may be able to modulate tumor cell behavior and enhance immune cell infiltration, potentially leading to improved patient outcomes.

However, a deeper understanding of the underlying mechanisms governing ephrin-immune interactions is essential to fully realize the therapeutic potential of this strategy. Future research should focus on identifying specific ephrin-mediated signaling pathways that drive tumor progression and immune evasion. By unraveling these mechanisms, we can develop more targeted and effective therapeutic interventions.

## Discussion

Eph/ephrin signaling plays a multifaceted role in skin cancers, influencing tumor cell behavior in various ways (**Table 2**). In keratinocytes, this signaling pathway helps maintain epidermal homeostasis by regulating proliferation, differentiation, and adhesion. However, in cancer, Eph/ephrin signaling can become dysregulated, promoting tumorigenesis, angiogenesis, and metastasis.

**Table 2:** Summary of the key Eph receptors and their specific effects related to skin cancer.

Eph Receptor (Ligand)	Regulation in Skin Cancer	Types of Skin Cancer Involved	Effects of Eph Receptor Regulation in Skin Cancer
EphA2 (ephrin-A1)	Upregulated	Malignant melanoma	- Promotes metastasis, immunosuppression, and cancer cell survival via Akt and ERK signaling. - Enhances migration, invasiveness, angiogenesis, and resistance to vemurafenib.
		SCC	- Increases lymph node metastasis and clinical stage. - Associated with decreased patient survival.
EphA3	Upregulated	Malignant melanoma	- Enhances cancer cell proliferation through ERK1/2 and p38 MAPK signaling.
EphA7	Downregulated	BCC	- Loss of EphA7 may contribute to BCC progression, but mechanisms are less well-defined.
EphB2	Upregulated	SCC	- Promotes tumor growth, invasiveness, and migration. - Increases cancer cell proliferation, reduces apoptosis, and correlates with higher histopathological grade.
EphB4 (ephrin-B2)	Upregulated	Malignant melanoma	- Enhances cancer cell adhesion and metastasis.
		SCC	- Stimulates angiogenesis, supporting tumor vascularization.
EphB6	Downregulated	Malignant melanoma	- Loss of EphB6 is linked to increased metastasis.

Given the widespread expression of Eph receptors across both malignant and immune cell populations, therapeutic strategies must carefully balance efficacy against unintended immunomodulatory effects. Inhibiting EphA2 signaling in tumor cells may suppress proliferation and invasion, yet concurrently dampening EphA2 function in immune cells could reduce T-cell responsiveness. This underscores the importance of precision-targeted approaches, such as ligand-mimetic inhibitors or selective monoclonal antibodies, which can differentiate between tumor-promoting and immune-supportive Eph/ephrin interactions.

In SCC, EphB2 emerges as a key player in tumor progression, with its overexpression correlating with aggressive disease and poor prognosis. Targeting EphB2 and related pathways could provide a therapeutic benefit, particularly in patients with high-risk or unresectable SCC. Similarly, EphA2 plays a pivotal role in survival, resistance to therapy, and metastasis in melanoma. EphA2’s overexpression in metastatic melanoma cells presents an attractive target for therapeutic intervention. Furthermore, the differential expression of other Eph receptors, such as EphB4 and EphB6, highlights the complexity of Eph/ephrin signaling in melanoma.

While significant progress has been made in understanding the role of Eph/ephrin signaling in skin cancers, several questions remain. For example, the mechanisms by which ephrin-A1 influences melanoma metastasis require further investigation. Additionally, the interaction between Eph/ephrin signaling and the TME, particularly with immune cells like macrophages, presents an area of active research that could yield new insights into tumor progression and treatment resistance.

**Figure 3:**
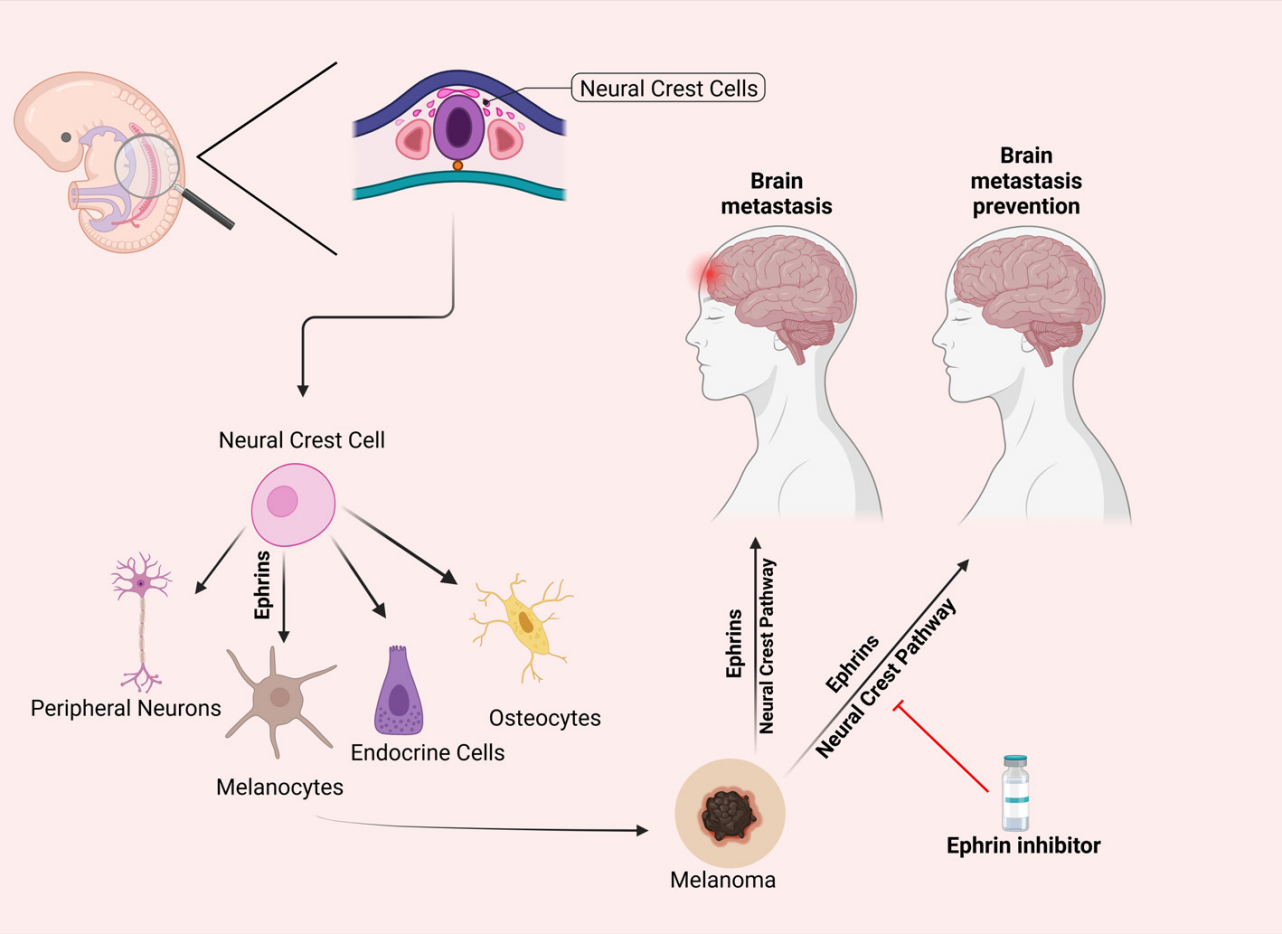
Differentiation of Neural Crest Cells and the Hypothesized Role of Ephrins in Preventing Melanoma Metastasis. This figure illustrates the differentiation of neural crest cells into peripheral neurons, melanocytes, endocrine cells, and osteocytes via ephrin signaling. The right panel proposes a hypothesis that inhibiting the ephrin pathway (indicated by the red line) may prevent melanoma metastasis to the brain by blocking pathways associated with neural crest cell migration. This potential preventative effect remains a subject of ongoing research. Parts of this figure were generated using BioRender.

Melanocytes originate embryonically from neural crest cells, a highly migratory cell population. The work of Krull et al. has demonstrated the critical role of the Eph/ephrin family, particularly the EphB subclass, in guiding the migration of neural crest cells during development (72). Building on this, it is plausible to hypothesize that dysregulated Eph/ephrin signaling in melanoma, a malignancy of melanocytes, could promote metastasis to the brain, a frequent and devastating metastatic site for melanoma. Since neural crest cells are known to follow specific migratory pathways under the influence of Eph/ephrin signaling (73), altered activation of these pathways in melanoma could enhance the tumor cells’ ability to invade the central nervous system (**Figure 3**).

This potential reactivation or hijacking of EphB-mediated migratory routes by melanoma cells offers a mechanistic explanation for the preferential spread of melanoma to neural tissues. Understanding this relationship could provide new insights into the metastatic behavior of melanoma and highlight Eph/ephrin signaling as a possible therapeutic target to prevent or limit brain metastasis.

While surgical excision remains the mainstay of skin cancer treatment, metastatic disease and unresectable tumors require alternative interventions. Identifying new molecular pathways for targeted therapies is a vital driver of cancer research. Eph/ephrin signaling represents a crucial yet complex pathway in the progression and metastasis of skin cancers. From maintaining normal epidermal homeostasis to driving metastatic spread in malignancies such as SCC and melanoma, this signaling network offers promising avenues for targeted therapy. EphA2, EphB2, and EphB4 stand out as critical therapeutic targets, especially in cases of aggressive or metastatic disease. In melanoma, the hypothesis of Eph/ephrin signaling to reactivate neural crest migratory pathways and contribute to brain metastasis provides a novel mechanistic insight that could inform future treatment strategies.

However, the dual nature of Eph/ephrin signaling—where it can act as both a tumor promoter and suppressor depending on the context—requires a nuanced approach to therapeutic targeting. Combining Eph-targeted therapies with conventional treatments, such as BRAF inhibitors, radiotherapy or immunotherapy, could yield improved outcomes, especially for patients with treatment-resistant or metastatic tumors.

In conclusion, further research into the specific roles of Eph receptors and ephrin ligands in tumor angiogenesis and metastasis, along with ongoing clinical trials targeting this pathway, will be essential for translating these findings into effective treatments for skin cancer patients. The potential of Eph/ephrin-based therapies to limit metastasis makes them an exciting area of investigation with significant clinical implications.
